# Gender-Specific Inverse Associations Between Beans Intake, Serum Urate Levels, and Hyperuricemia: A Cross-Sectional Analysis Based on the Henan Rural Cohort Study

**DOI:** 10.3389/fnut.2020.593599

**Published:** 2021-01-21

**Authors:** Ningning Cui, Xiaokang Dong, Yuan Xue, Wei Liao, Xiaotian Liu, Yuqian Li, Jian Hou, Wenqian Huo, Linlin Li, Zhenxing Mao, Zhaohui Zheng, Chongjian Wang

**Affiliations:** ^1^Department of Epidemiology and Biostatistics, College of Public Health, Zhengzhou University, Zhengzhou, China; ^2^Department of Nutrition and Food Hygiene, College of Public Health, Zhengzhou University, Zhengzhou, China; ^3^Department of Rheumatology, The First Affiliated Hospital of Zhengzhou University, Zhengzhou, China

**Keywords:** beans intake, serum urate, hyperuricemia, rural population, gender-specific

## Abstract

**Background and Aims:** Beans are rich in purines, which are important substances that lead to elevated serum urate, especially exogenous purines. Few studies were conducted to assess the relationship between beans intake and serum urate or hyperuricemia, especially in rural people. The purpose of this study was to validate the association by gender in the rural Chinese population.

**Methods:** A total of 38,855 participants aged 18–79 years old were enrolled from the Henan Rural Cohort Study (Registration number: ChiCTR-OOC-15006699). Dietary data were collected using a validated food frequency questionnaire (FFQ). Linear regression models and logistic regression models were used to examine the associations between beans intake and serum urate levels or hyperuricemia. Restricted cubic spline regression was performed to display the dose–response relationship.

**Results:** In multivariate-adjusted linear regression, an inverse correlation was found between beans intake and serum urate level (the highest quartile Q4 vs. the bottom quartile Q1) in both men (*P* = 0.008) and women (*P* < 0.001). Per 10-g increment in beans intake was associated with 0.30 μmol/L decreased concentration of serum urate in men and 0.71 μmol/L in women. The multivariate-adjusted odds ratios (ORs) of hyperuricemia were 0.83 (0.71, 0.97) in men and 0.73 (0.63, 0.84) in women (Q4 vs. Q1). Per 10-g increment in beans intake created a 1% decreased risk of hyperuricemia in men and 3% in women. The cubic spline suggested a risk reduction for hyperuricemia with increasing intake of beans.

**Conclusion:** A higher beans intake was associated with a lower serum urate level and a reduced risk of hyperuricemia in both sexes, and the association was more pronounced in women.

## Introduction

As for 2014, the prevalence of hyperuricemia in mainland China has reached 13.3% (1); it is therefore safe to assume that hyperuricemia affects no <180 million people in China based on the increasing prevalence. Notably, hyperuricemia has been illustrated as a risk factor for many other diseases, for example, metabolic syndrome (2), gout (3), T2DM (4), hypertension and cardiovascular disease (5), dyslipidemia (6), as well as renal damage (7). Hyperuricemia and its complications have greatly increased the global burden of disease (8). Studies found that hyperuricemia was a metabolic disease, because of urate overproduction, underexcretion, or the combined effect of them (9). As for serum urate, the end product of purine, it was mainly converted by endogenous or dietary purine (10). An effective strategy is urgently needed to decrease the levels of serum urate for patients with hyperuricemia. Dietary factors and lifestyle were attached to an important concern in related studies owing to the characteristics of changeability (11–14).

For many years, a diet low in purine was recommended as a standard dietary pattern to maintain normal serum urate levels. Whether all purine-rich foods are risk factors of hyperuricemia is still debatable. Quite a few studies have informed that meat, animal sources of protein, seafood, alcohol, and other purine-rich foods contribute to increased serum urate (14, 15). Nevertheless, abundant studies have demonstrated that purine-rich vegetables were inversely associated with serum urate concentration (16–18). Beans are rich in purine, which is the major reason for doctors to restrict the intake of beans to patients with elevated uric acid in the past. To date, fewer studies have been performed to verify the correctness of this practice. Current experimental and epidemiological studies provide inconsistent evidence toward the association between beans and hyperuricemia. Many prior studies indicated beans intake had nothing to do with the increase of serum urate levels or even was associated with lower risk for hyperuricemia (14, 19–21). Simultaneously, certain studies held opposite views that beans intake was closely related to the increase of serum urate concentration (22, 23). The associations of beans intake and serum urate levels with hyperuricemia are yet inconsistent and further studies are indispensable to address this unclear issue.

Although several previous studies have been carried out to explore the relationship between beans intake and the prevalence of hyperuricemia, most of them were conducted in high-income countries or regions, or in urban population (14, 19). Up to now, studies in rural populations have remained unknown. In order to achieve the strategic goal of a healthy China by 2030, it has become an inevitable trend to pay more attention to the health of the rural population. Additionally, beans and soybean products are more common in the diet of rural population, compared to the animal-based dietary pattern of urban population. To address this gap, a large sample-size, cross-sectional survey was conducted to investigate the associations between beans intake, serum urate levels, and hyperuricemia in rural Chinese population from the Henan Rural Cohort Study.

## Materials and Methods

### Study Population

**Figure 1:**
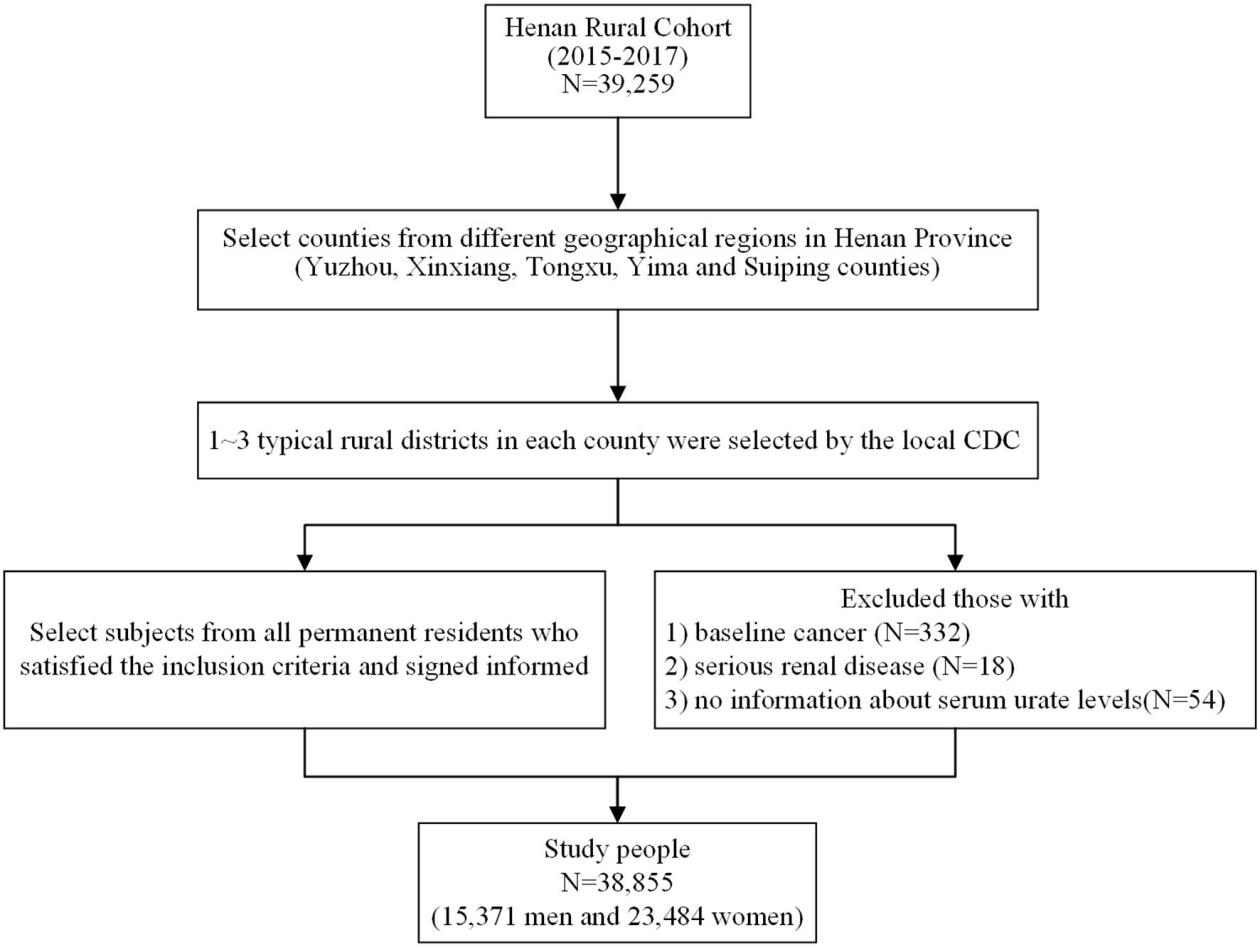
Flow chart showing inclusion of study participants from the initial cohort of 39,259 participants in the Henan Rural Cohort Study, 2015–2017.

The study subjects were derived from the Henan Rural Cohort Study performed in the rural areas in Henan province from July 2015 to September 2017, which has been registered before the start of participant enrollment in the Chinese Clinical Trial Register (Registration number: ChiCTR-OOC-15006699). Details of the study design, methods, and participants' characteristics have been previously described (24, 25). In short, the Henan Rural Cohort Study is an ongoing prospective cohort study that enlisted 39,259 cohort members aged 18–79 years for baseline data. To better validate the relationship between beans consumption and the prevalence of hyperuricemia, 332 subjects with diagnosed cancers, 18 subjects with severe renal disease, and 54 subjects without information about serum urate levels were excluded; 38,855 subjects were enrolled in the present analysis. The flow chart is presented in Figure 1. The study was conducted according to the Declaration of Helsinki guidelines, and the protocol of this study was approved by the “Zhengzhou University Life Science Ethics Committee” [Code: (2015) MEC (S128)]. Informed consent was obtained from each participant.

### Dietary Assessment and Beans Intake

At baseline, a semi-quantitative 13-item food frequency questionnaire (FFQ) was utilized to identify dietary conditions in the rural Chinese population. All data for the FFQ were obtained by experienced interviewers during a face-to-face visit. These foods were covered under the categories of staple foods, livestock, poultry, fish, eggs, milk, fruits, vegetables, beans, nuts, pickles, cereal, and animal oil. Respondents were asked to report the quantity and frequency of each specific item during the previous year. The quantity of food consumed (in kilograms and grams) was assessed by interviewees' statements under five frequencies of consumption (never, day, week, month, and year) using a card with samples for typical diets and portion sizes as visual aids. Then, food intake was calculated by multiplying the quantity consumed by the frequency. Dietary nutrient (carbohydrate, protein, and total fat) intake was calculated based on the China Food Composition Table 2004 (26). Beans intake in this study was identified as the total consumption of all beans commonly eaten by local people such as soybean, lentil, red bean, mung bean, black bean, pea, etc. Beans intake was divided into four groups according to the quartiles of sex-specific distribution: <7.14, 7.14–21.43, 21.44–50.00, and ≥50.01 g/day for men; <4.11, 4.11–16.67, 16.68–42.86, and ≥42.87 g/day for women.

The reproducibility and validity of the FFQ were validated among 295 and 123 cohort members, respectively, using repeat measurements and three 24-h recall assessments (24). The correlation coefficient of reproducibility and validity was 0.22 and 0.13 for beans intake, respectively (27).

### Assessment of Potential Covariates

A standardized questionnaire was utilized to retrieve other information from participants by well-trained investigators, including general demographic characteristics (name, sex, age, education levels, marital status, and income), lifestyles (smoking and drinking status, physical activity levels), family and personal disease history, etc. Taking high school as the boundary, education levels were divided into “ ≤ primary school” and “≥ junior school” grades. Smoking status was classified into current smoker (a person who smoked more than one cigarette per day in the past 6 months), ever smoker (a person who ever smoked), and never smoker. Alcohol drinking status was categorized into current drinking (a person who consumed 12 or more alcoholic drinks in the past year, whether spirits, beer, wine, or other forms of alcohol beverage), ever drinking (a person who ever drank), and never drinking. Electronic sphygmomanometer (Omron HEM-7071A, Japan) was used to measure systolic blood pressure (SBP) and diastolic blood pressure (DBP) in standard methods. SBP and DBP were both measured three times in a seated position with a 1-min interval before each measurement.

The venous blood samples were collected by professional medical staff from subjects who kept an overnight fast for at least 8 h. Subsequently, these samples were centrifuged for 10 min using 3000 rpm at 4°C to get the serum, which was stored in a −80°C cryogenic refrigerator. ROCHE Cobas C501 automatic biochemical analyzer was used to measure serum urate levels with the enzymatic colorimetric method. A modified hexokinase enzymatic method was applied to detect the concentration of fasting plasma glucose (FPG). The ROCHE Cobas C501 automatic biochemical analyzer (Cobas C501, Roche Diagnostics GmbH) was used to measure total cholesterol (TC), high-density lipoprotein cholesterol (HDL-C), low-density lipoprotein cholesterol (LDL-C), triglycerides (TG), and serum creatinine (Cr).

### Definition of Clinical Outcomes

A serum urate >7.0 mg/dl (417 μmol/L) in men and >6.0 mg/dl (357 μmol/L) in women was universally acknowledged as hyperuricemia (28). According to the International Physical Activity Questionnaire (IPAQ 2001), physical activity was divided into low, moderate, and high levels. Subjects who were diagnosed by physicians as having diabetes, those whose fasting blood glucose ≥7.0 mmol/L, or those who were currently taking insulin or oral hypoglycemic agents were considered to have diabetes. Hypertension was defined as a value of SBP ≥140 mmHg and/or DBP ≥90 mmHg; those who were taking antihypertensive medications in the course of the previous 2 weeks were considered to have hypertension. Anybody who reported physician-diagnosed dyslipidemia, or whose TC level ≥6.22 mmol/L, or LDL cholesterol ≥4.14 mmol/L, or a low HDL cholesterol <1.04 mmol/L, or TG ≥2.26 mmol/L, or those who were taking anti-dyslipidemia medications in the past 2 weeks were considered to have dyslipidemia. Family history of gout was defined as either parents or siblings of the subjects having a history of gout.

### Statistical Analysis

All statistical analysis was conducted by gender independently. Analysis of variance (ANOVA) and chi-square test were utilized to analyze baseline characteristics of study subjects, which applied to continuous data and qualitative variables, respectively. The outcomes were presented as means ± SD (continuous data) and percentages (qualitative variables). Previous studies have noticed that the demographic characteristics and lifestyle were important risk factors for the prevalence of hyperuricemia; to our knowledge, the frequency, and amount of one food would affect the other food consumption. Therefore, multivariate-adjusted regression analysis was performed to ensure the authenticity and comparability of the results. Using the lowest quartile as the reference group, multiple linear regressions were used to estimate the correlation between beans intake and serum urate level assessed by the regression coefficient (β) with its 95% confidence interval (CI); multivariate logistic regression analysis was performed to explore the association between beans intake and the risk of hyperuricemia evaluated by odds ratio (OR) and its CI. The interaction effect between gender–beans intake and hyperuricemia was also conducted using logistic regression method. Based on previous studies on hyperuricemia (12, 14, 26), some potential covariates were selected and three regression models were employed. The directed acyclic graphs (DAGs) were used to present the confounders selected (Supplementary Figure 3). Model 1 was a crude unadjusted model, and model 2 was adjusted for age, education levels, marital status, average monthly income, smoking status, alcohol drinking, physical activity, waist circumference, red meat, white meat, fish, milk, vegetable intake, fruit intake, gout, family history of gout, and energy intake. Multivariable model 3 was additionally adjusted for diabetes mellitus, hypertension, and dyslipidemia status. The *P*-value for trend was performed regarding beans intake as a continuous variable, adjusting all confounders aforementioned. To minimize the error caused by information loss in the process of shifting continuous variable (beans intake) into categorical variable, the fitting restricted cubic spline logistic regression was performed to monitor the dynamic change of hyperuricemia risk with the change of beans intake and identify the dose–response associations of beans intake and hyperuricemia risk.

All statistical analyses were carried out using the SPSS 21.0 and the SAS V.9.1 (SAS Institute) software. A *P* < 0.05 (two-sided test) was viewed as statistically significant in this study.

## Results

**Figure 2:**
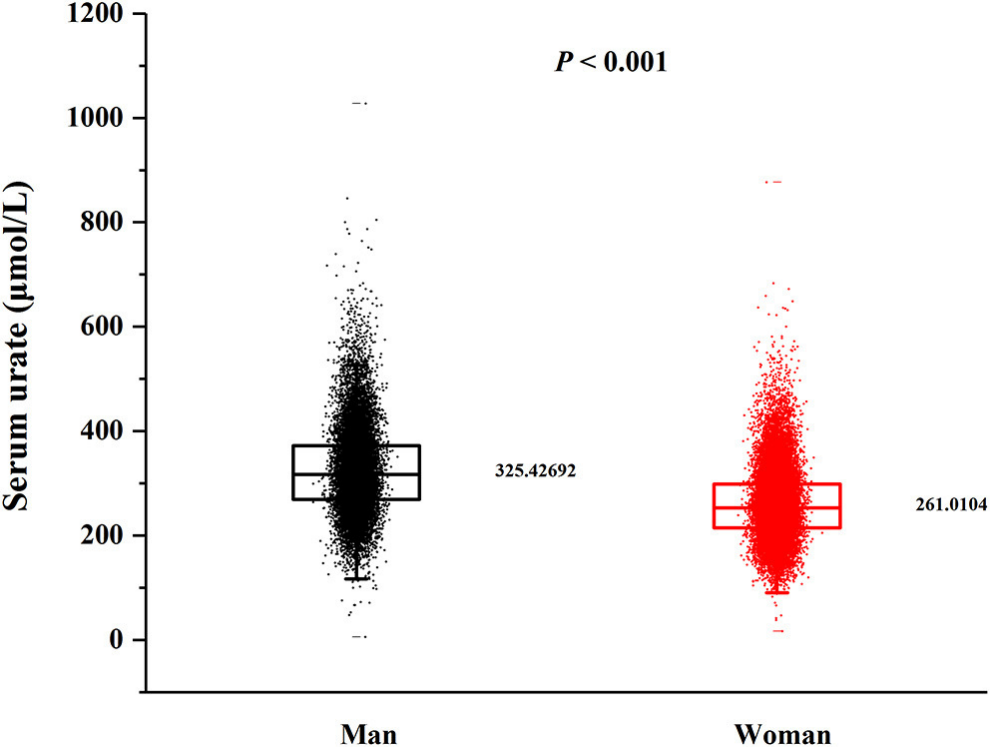
Distribution of serum urate by gender. *P*-value was calculated by Student's *t-*test.

**Figure 3:**
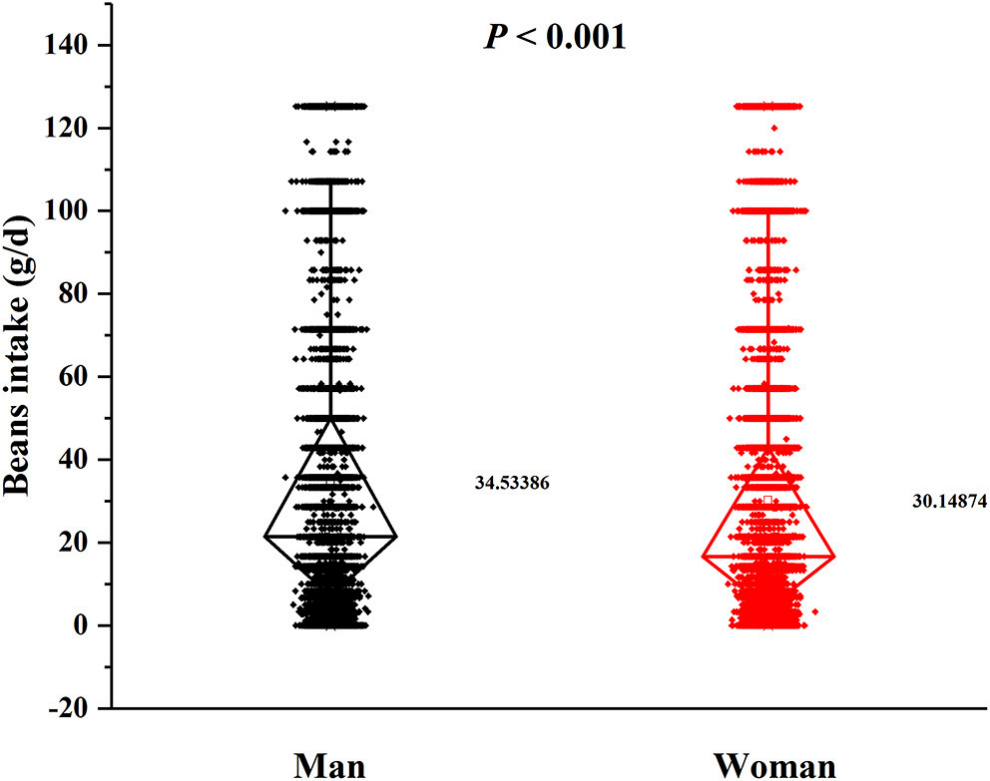
Distribution of beans intake by gender. *P*-value was calculated by Student's *t-*test.

In this cross-sectional study, a total number of 38,855 (15,371 men and 23,484 women) rural adults were included. A significant difference of serum urate levels and the distribution of beans intake by gender was observed (Figures 2, 3), thus, the analysis of this study was performed across gender.

### Characteristics of Study Population

Table 1 presents the gender-specific baseline characteristics of the study population by quartile of total beans intake. Significant differences in total beans consumption were shown in both men and women (*P* < 0.001). Individuals located in the highest quartile of beans consumption were prone to be younger and have higher education level and income. A significant difference was found in smoking status and disease history (diabetes, hypertension, and dyslipidemia) among the four levels in men but not in women. Moreover, participants with higher consumption of total beans have better dietary patterns characterized by consuming more milk, vegetables, and fruits.

**Table 1:** Baseline characteristics of the study population by quartile of total beans intake in men and women (*N* = 38,855)^a^.

**Variables**	**Men (*****N*** **= 15,371)**									**Women (*****N*** **= 23,484)**								
	**Q1 (*****N*** **= 4,247) <7.14 g/day**		**Q2 (*****N*** **= 4,111) 7.14–21.43 g/day**		**Q3 (*****N*** **= 3,694) 21.44–50.00 g/day**		**Q4 (*****N*** **= 3,319)** ≥**50.01 g/day**		***P^b^***	**Q1 (*****N*** **= 5,895) <4.11 g/day**		**Q2 (*****N*** **= 6,741) 4.11–16.67 g/day**		**Q3 (*****N*** **= 4,931) 16.68–42.86 g/day**		**Q4 (*****N*** **= 5,917) ≥42.87 g/day**		***P^b^***
	**Mean**	***SD***	**Mean**	***SD***	**Mean**	***SD***	**Mean**	***SD***		**Mean**	***SD***	**Mean**	***SD***	**Mean**	***SD***	**Mean**	***SD***	
Beans (g/day)	2.4	2.8	15.3	4.0	36.6	7.8	97.1	23.6	<0.001	1.0	1.4	11.6	3.9	28.6	5.6	81.6	30.4	<0.001
Serum urate (μmol/L)	321.1	81.6	325.0	81.2	329.3	2.9	327.3	81.4	<0.001	263.5	67.9	263.6	67.8	261.2	67.2	259.0	65.3	0.002
Age (years)	58.2	12.2	57.0	12.2	55.4	12.4	55.3	12.2	<0.001	56.4	11.8	55.3	11.8	54.3	12.2	53.4	12.5	<0.001
Education (%)									<0.001									<0.001
≤Primary school	43.0		32.9		29.6		27.6			62.2		53.6		48.2		43.0		
≥Junior school	57.0		67.1		70.4		72.4			37.8		46.4		51.8		57.0		
Marital status (%)									<0.001									<0.001
Married/cohabiting	87.0		90.5		91.3		91.6			86.4		90.1		90.4		91.5		
Widowed/single/ divorced/separated	13.0		9.5		8.7		8.4			13.6		9.9		9.6		8.5		
Average monthly individual income (%)									<0.001									<0.001
<500 RMB	45.1		35.1		32.4		29.9			43.3		35.2		32.3		29.9		
500–1,000 RMB	30.4		34.3		32.3		30.1			31.0		35.9		35.2		32.5		
≥1,000 RMB	24.5		30.6		35.3		40.0			25.7		28.9		32.5		37.6		
Smoking status (%)									0.008									0.517
Non-smoker	32.8		32.5		30.0		30.8			99.5		99.7		99.7		99.6		
Ex-smoker	20.1		20.8		20.8		19.0			0.1		0.1		0.1		0.1		
Smoker	47.0		46.6		49.2		50.2			0.4		0.2		0.2		0.3		
Drinking status (%)									<0.001									0.053
Non-drinker	55.0		45.9		43.1		41.8			97.2		97.1		97.5		96.8		
Ex-drinker	11.3		11.5		11.2		11.1			0.4		0.2		0.3		0.2		
Drinker	33.6		42.6		45.7		47.1			2.4		2.7		2.2		3.0		
Physical activity (%)									0.213									<0.001
Low	35.8		35.1		37.0		34.4			29.7		31.4		28.5		30.9		
Moderate	27.9		27.2		127.6		28.7			43.3		42.5		45.6		45.7		
High	36.3		37.8		35.5		36.9			27.0		26.1		25.9		23.5		
Diabetes mellitus (%)									0.001									0.467
No	92.2		91.0		90.5		89.6			90.4		90.1		90.9		90.0		
Yes	7.8		9.0		9.5		10.4			9.6		9.9		9.1		10.0		
Hypertension (%)									0.016									0.114
No	74.7		72.2		71.8		72.4			74.3		73.0		73.6		74.8		
Yes	25.3		27.8		28.2		27.6			25.7		27.0		26.4		25.2		
DYS (%)									<0.001									0.110
No	62.9		59.6		56.9		59.5			63.6		63.4		64.6		65.2		
Yes	37.1		40.4		43.1		40.5			36.4		36.6		35.4		34.8		
WC (cm)	83.9	10.6	85.4	10.5	86.7	10.5	86.8	10.3	<0.001	82.7	10.3	82.9	10.2	83.4	10.1	83.4	10.0	<0.001
Red meat (g/day)	27.6	34.3	34.3	35.5	40.67	37.2	50.0	43.0	<0.001	20.6	29.3	23.0	28.1	29.0	31.6	35.5	36.7	<0.001
White meat (g/day)	10.6	14.4	13.5	14.9	16.9	16.8	20.6	18.9	<0.001	8.2	12.5	9.9	12.8	13.3	15.2	15.7	17.0	<0.001
Fish (g/day)	3.0	4.8	4.2	5.4	5.0	5.9	5.8	6.5	<0.001	2.1	4.0	2.9	4.4	3.8	5.3	4.5	5.8	<0.001
Milk (g/day)	9.0	18.2	12.0	20.2	13.2	21.1	14.7	22.2	<0.001	7.8	17.4	11.4	19.8	13.1	21.2	16.2	22.9	<0.001
Vegetable intake (g/day)	318.1	195.5	318.6	176.2	332.3	176.6	351.9	203.6	<0.001	308.6	183.1	298.4	165.8	313.4	164.4	321.2	185.1	<0.001
Fruit intake (g/day)	107.9	131.3	122.9	132.8	136.1	133.6	163.2	146.6	<0.001	118.7	133.4	134.1	130.9	154.5	1312	181.2	142.5	<0.001
Energy intake (kJ)	2538.8	672.3	2616.5	655.2	2740.5	642.7	3090.8	712.4	<0.001	2115.9	569.3	2152.3	525.8	2284.4	529.5	2557.0	612.5	<0.001
Gout (%)									0.178									0.849
No	99.7		99.8		99.5		99.5			99.6		99.6		99.7		99.6		
Yes	0.3		0.2		0.5		0.5			0.4		0.4		0.3		0.4		
Family history of gout (%)									0.481									0.441
No	99.9		99.9		99.8		99.8			99.8		99.7		99.7		99.7		
Yes	0.1		0.1		0.2		0.2			0.2		0.3		0.3		0.3		

*DYS, dyslipidemia; Q1–Q4, quartile of beans intake (from lowest to highest); SD, standard deviations; WC, waist circumference*.

a *Continuous variables were presented as mean ± SD (n); categorical variables are shown as n (%)*.

b *P-value was calculated by ANOVA for continuous variables and the χ^2^-test for categorical variables*.

### Relationship Between Beans Intake and Serum Urate

Multiple linear regression analysis showed a statistically significant association between beans intake and serum urate levels (Table 2). After adjusting for multiple potential confounders (Model 3), a significantly inverse association was observed between total beans consumption and serum urate levels. Compared with the first quartile, the β and 95% CI of Q1, Q2, and Q3 were −2.41 (−5.67, 0.85), −3.75 (−7.51, −0.35), and −4.67 (−8.32, −1.02) in men (*P*_fortrend_ = 0.008) and −3.52 (−5.75, −1.28), −6.41 (−8.86, −3.96), and −8.26 (−10.71, −5.81) in women (*P*_fortrend_ < 0.001). Per 10-g increment in beans intake was significantly associated with 0.30 μmol/L decreased concentration of serum urate in men and 0.71 μmol/L in women.

**Table 2:** Multivariate-adjusted β-coefficients and 95% confidence interval for serum urate levels according to quartiles of total beans intake.

**Variables**	*****β***** **(95% CI)**		
	**Model 1**	**Model 2**	**Model 3**
**Men**			
Q1	0 (Ref.)	0 (Ref.)	0 (Ref.)
Q2	3.93 (0.42, 7.44)	−2.31 (−5.63, 1.01)	−2.41 (−5.67, 0.85)
Q3	8.28 (4.67, 11.89)	−3.67 (−7.13, −0.20)	−3.75 (−7.15, −0.35)
Q4	6.20 (2.49, 9.91)	−5.35 (−9.07, −1.64)	−4.67 (−8.32, −1.02)
Per 10-g increment	0.62 (0.27, 0.96)	−0.37 (−0.72, −0.02)	−0.300 (−0.64, −0.04)
*P*_trend_	<0.001	0.003	0.008
**Women**			
Q1	0 (Ref.)	0 (Ref.)	0 (Ref.)
Q2	−2.41 (−4.74, −0.07)	−3.32 (−5.60, −1.05)	−3.52 (−5.75, −1.28)
Q3	−3.54 (−6.07, −1.02)	−6.48 (−8.97, −3.99)	−6.41 (−8.86, −3.96)
Q4	−4.62 (−7.03, −2.21)	−8.51 (−11.00, −6.02)	−8.26 (−10.71, −5.81)
Per 10-g increment	−0.42 (−0.67, −0.18)	−0.78 (−1.03, −0.52)	−0.71 (−0.96, −0.46)
*P*_trend_	<0.001	<0.001	<0.001

*CI, confidence interval; Ref, reference*.

*Model 1: unadjusted*.

*Model 2: adjusted for age, education, marital status, average monthly individual income, smoking status, drinking status, physical activity, waist circumference, red meat, white meat, fish, milk, vegetable intake, fruit intake, energy intake, gout, and family history of gout*.

*Model 3: additionally adjusted for diabetes mellitus, hypertension, and dyslipidemia*.

*P_trend_ was performed using the quartiles of total beans intake as a continuous variable in the linear regression model*.

### Relationship Between Beans Intake and Hyperuricemia

Multivariate-adjusted ORs and 95% CI for hyperuricemia according to quartile of beans intake are depicted in Table 3. After adjusting for all potential confounders (Model 3), more intake of beans presented beneficial effects. Individuals in the highest quartile of beans intake were 17 and 25% less likely to have hyperuricemia than those in the lowest quartile for men and women, respectively. Compared with the lowest quartile of beans intake, the ORs and 95% CI for hyperuricemia were 0.93 (0.81, 1.07), 0.88 (0.76, 1.01), and 0.83 (0.71, 0.97) in men (*P*_fortrend_ = 0.015) and 0.91 (0.81, 1.04), 0.85 (0.74, 0.97), and 0.73 (0.63, 0.84) in women (*P*_fortrend_ < 0.001), respectively, with each increasing quartile. Per 10-g increment in beans intake was significantly associated with a 1% decreased risk of hyperuricemia in men and 3% in women. In addition, a significant interactive effect of gender and beans intake on hyperuricemia was observed (*P* = 0.028).

**Table 3:** Multivariate-adjusted OR and 95% CI for hyperuricemia according to quartile of total beans intake.

**Variables**	**OR (95% CI)**		
	**Model 1**	**Model 2**	**Model 3**
**Men**			
Q1	1.00 (Ref.)	1.00 (Ref.)	1.00 (Ref.)
Q2	1.08 (0.95, 1.23)	0.94 (0.82, 1.08)	0.93 (0.81, 1.07)
Q3	1.19 (1.04, 1.35)	0.89 (0.77, 1.03)	0.88 (0.76, 1.01)
Q4	1.12 (0.97, 1.28)	0.83 (0.71, 0.97)	0.83 (0.71, 0.97)
Per 10-g increment	1.02 (1.00, 1.03)	0.99 (0.98, 1.00)	0.99 (0.98, 1.01)
*P*_trend_	0.04	0.012	0.015
**Women**			
Q1	1.00 (Ref.)	1.00 (Ref.)	1.00 (Ref.)
Q2	0.93 (0.83, 1.05)	0.92 (0.81, 1.04)	0.91 (0.81, 1.04)
Q3	0.91 (0.80, 1.04)	0.84 (0.73, 0.97)	0.85 (0.74, 0.97)
Q4	0.81 (0.71, 0.92)	0.73 (0.64, 0.84)	0.73 (0.63, 0.84)
Per 10-g increment	0.98 (0.97, 0.99)	0.97 (0.96, 0.97)	0.97 (0.96, 0.99)
*P*_trend_	<0.001	<0.001	<0.001
*P*_beansintake−gender_	<0.001	0.016	0.028

*OR, odds ratio; CI, confidence interval; Ref, reference*.

*Model 1: unadjusted*.

*Model 2: adjusted for age, education, marital status, average monthly individual income, smoking status, drinking status, physical activity, waist circumference, red meat, white meat, fish, milk, vegetable intake, fruit intake, energy intake, gout, and family history of gout*.

*Model 3: additionally adjusted for diabetes mellitus, hypertension, and dyslipidemia*.

*P_trend_ was performed using the quartile of total beans intake as a continuous variable in the logistic regression model*.

*P_beansintake−gender_ was performed using the interaction term, beans intake-gender, as a continuous variable in the logistic regression model*.

**Figure 4:**
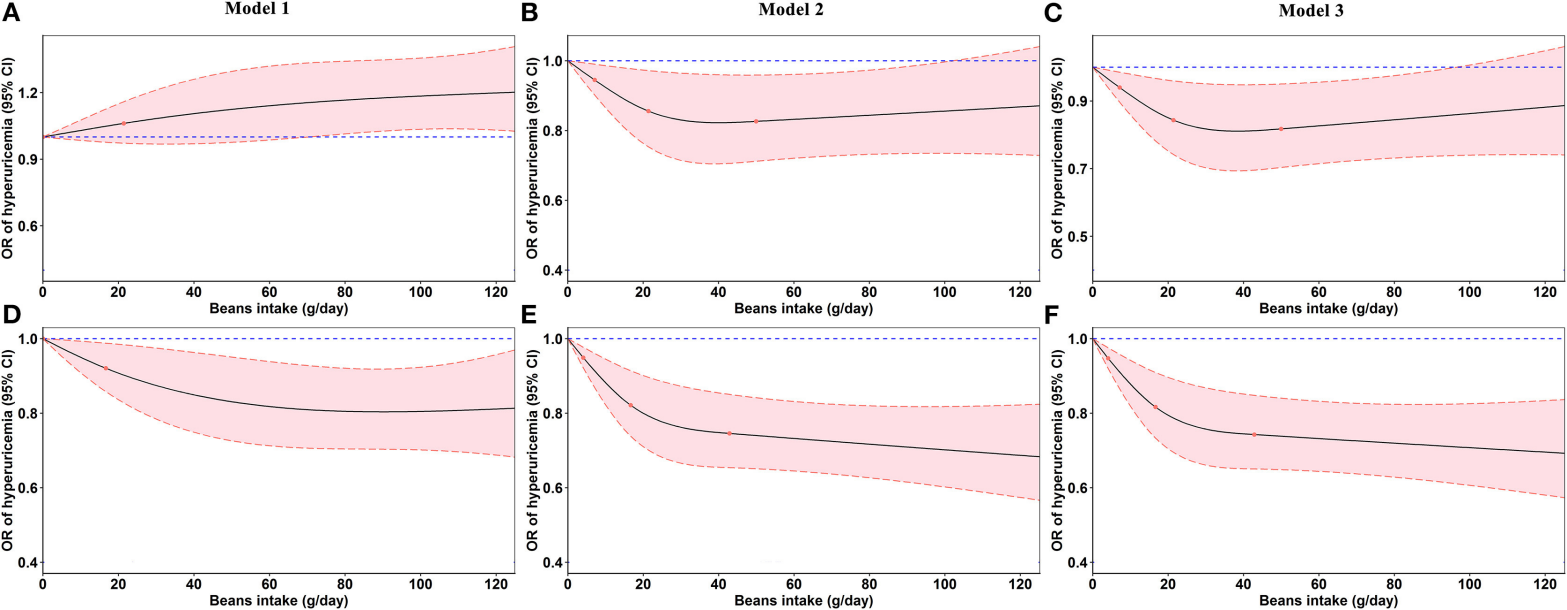
Odds ratios (ORs, solid lines) and 95% confidence intervals (CIs, dashed lines) of beans intake for hyperuricemia from restricted cubic splines. **(A–C)** Odds ratios and 95% confidence intervals of beans intake for hyperuricemia in men. **(D–F)** Odds ratios and 95% confidence intervals of beans intake for hyperuricemia in women. Model 2 was adjusted for age, education, marital status, average monthly individual income, smoking status, drinking status, physical activity, waist circumference, red meat, white meat, fish, milk, vegetable intake, fruit intake, energy intake, gout, and family history of gout. Model 3 was additionally adjusted for diabetes mellitus, hypertension, and dyslipidemia.

In Figure 4, the restricted cubic splines showed that the OR of hyperuricemia significantly decreased with the increasing beans intake after adjusting for confounding factors, which was more evident in women (Figures 4E,F).

## Discussion

In the large-scale rural Chinese population-based cross-sectional study, the main findings suggest that habitual consumption of beans was inversely associated with serum urate levels and hyperuricemia risk. The associations were more pronounced in women and a significant interactive effect of gender and beans intake on hyperuricemia was observed. Additionally, an inverse dose–response relationship was also found between beans intake and the risk of hyperuricemia.

Few studies on the association between beans intake and serum urate levels or hyperuricemia are known, and by far, there has been no study on the rural population. Although beans intake was anecdotally considered a protective factor for serum urate reduction and hyperuricemia, more large-scale studies are requisite to formally test this hypothesis and quantify the magnitude of the association. Until now, attitudes to the association between beans intake and hyperuricemia are inconsistent among experimental and epidemiological studies. An ongoing, 6-year randomized clinical trial (the PREDIMED-Plus study) (19) indicated that non-soy legumes were inversely associated with serum urate levels and hyperuricemia prevalence, which was in line with the current study based on the Henan Rural Cohort Study. A study in the framework of Jinchang Cohort indicated that weekly beans intake ≥0.25 kg was associated with a lower risk for gout (HR = 0.528, 95% CI: 0.345–0.808) (29). Not only beans but also SVEs supplements (Soy Vinegar Extracts) have been shown to slow the development of hyperuricemia (30). On the contrary, a study implemented among 178 infants suggested that soy formula affected urate excretion and increased serum urate concentrations (23). The other study performed among Chinese men also affirmed that soybeans, soybean milk, and soy powder considerably increased serum urate levels (31).

In order to overcome the defects of logistic regression models, a restricted cubic spline model was fitted to present the effects of beans intake on hyperuricemia more intuitively. In accordance with the regression analysis, beans intake had a positive effect on lowering serum urate and reducing hyperuricemia risk. However, the effect was more pronounced in women. Our results were similar with Liu et al.'s (32) previous study, which also found significantly inverse association between greater consumption of soy food and the presence of hyperuricemia in women, but not in men. As for the difference, it may be ascribed to the interactive effect of gender and beans intake (Table 3). No plausible explanation was found for the observed differences between men and women. Different lifestyles and eating habits, which are presented in the baseline data, may be responsible for the divergent outcomes. In addition, the genetic (33) and hormonal (34) differences between genders as well as the causes cannot be ignored, which promote the serum urate excretion or prevent the development of hyperuricemia. Nevertheless, further studies are warranted in the future to detect these possible causes for the differences by sex.

In recent years, it has been hot to search for the association of purine-rich food with serum urate levels and hyperuricemia. Although beans are purine-rich food, the associations were not as expected in many previous studies (4, 19, 20) and our findings. A meta-analysis and systematic review conducted by Li et al. (35) included 19 prospective cohort or cross-sectional studies, also drawing the conclusion that soy food intake was negatively associated with gout (OR: 0.85; 95% CI: 0.76–0.96) and hyperuricemia (OR: 0.70; 95% CI: 0.56–0.88).

There are some biochemical expositions for the unexpected phenomenon. Phytic acid was found in substantial amounts in beans, which demonstrated a health-related beneficial role in controlling post-prandial serum urate (*P* < 0.05) (36). Moreover, our findings may be partly ascribed to beans' affluent content of polyphenols, mainly phenolic acids, and flavonoids, which were convinced of the anti-hyperuricemic effect (37). In the study of Spanou C published in 2012 (38), it was informed that flavonoid glycosides isolated from *Vicia faba* and *Lotus edulis* could inhibit the activity of xanthine oxidase, a key enzyme in purine metabolism. The same result was inspected next year (39). Furthermore, purine content in food had a change after cooking or processing (40). Consumption of soy products was significantly associated with lower risk of hyperuricemia; however, null association was found in unprocessed soy beans (14). In addition, plant-derived foods, especially nuts and beans, were commonly considered “good for health” with the preferred source of protein for gout patients (15).

### Strengths and Limitations

To the best of our knowledge, this is by far the largest study to investigate the association between beans intake and serum urate levels and hyperuricemia in a rural Chinese population. The strengths of the present study are its large sample size, strict quality assurance and control, a validated FFQ, and adjustment of a number of potential confounders.

There are several limitations that should not be overlooked in this study. Firstly, the present study was cross-sectional in design, which was limited to predicting the cause–effect relationship. Since this study is a population-level study, it is limited in terms of individual personalized advice. Therefore, further prospective studies are required to verify our findings. Secondly, the use of FFQ to assess the intake of beans was not as accurate as the 3-day dietary review. However, the FFQ is designed to mean quantity and frequency of food consumed over the previous year. It is not possible to obtain accurate diet data based on only one 3-day dietary survey (three 24DR), especially for those food groups that are eaten on special days. Additionally, the FFQ just contained 13 items that may ignore some foods' effect on serum urate. During the data collection, there was no detailed record of each type of bean intake, so it was not available to determine which kinds of beans consumed were responsible for the decreased serum urate. In addition, the analysis did not verify which component in beans caused the decrease in serum urate concentration. Thirdly, a rich range of risk factors attained have been adjusted to better the present study, but some unknown or unmeasured factors might have been existent, leading the result to unexpected directions. Finally, as the subjects all come from Henan province located in the middle area of China, the applicability of the results in other populations remains to be studied. However, given that Henan has a large rural population that accounts for 10% of rural Chinese population, the findings are representative. Otherwise, studies that attached importance to rural populations are scarce, so the current study will provide authentic evidence across the effect of beans on the control of serum urate levels and hyperuricemia in rural individuals.

## Conclusions

In conclusion, beans intake was associated with lower serum urate levels and decreased prevalence of hyperuricemia among the rural Chinese population. The associations were more pronounced in women ascribed to the interactive effect of gender and beans intake on hyperuricemia. Our findings address the data gap and can be used to further improve dietary guidance for people with urate metabolism disorder.

## Data Availability Statement

The raw data supporting the conclusions of this article will be made available by the authors, without undue reservation.

## Ethics Statement

The studies involving human participants were reviewed and approved by Zhengzhou University Life Science Ethics Committee. The patients/participants provided their written informed consent to participate in this study.

## Clinical Trial Registration

Before our study, the Henan Rural Cohort Study has been registered in the Chinese Clinical Trial Register (Registration number: ChiCTR-OOC-15006699).

## Author Contributions

CW and ZZ conceived and designed the study. XD, XL, YX, YL, JH, WH, LL, ZM, and ZZ coordinated data collection. NC, XD, and WL conducted the analyses. NC and XD wrote the manuscript. All authors have approved the final manuscript.

## Conflict of Interest

The authors declare that the research was conducted in the absence of any commercial or financial relationships that could be construed as a potential conflict of interest.
