# Supplementary material for: Gender-Specific Inverse Associations Between Beans Intake, Serum Urate Levels, and Hyperuricemia: A Cross-Sectional Analysis Based on the Henan Rural Cohort Study
Source: Front Nutr. 2021 Jan 21;7:593599. doi: 10.3389/fnut.2020.593599 (PMC7859095; doi:10.3389/fnut.2020.593599)
Supplement: Supplementary file 1 [file Data_Sheet_1.docx]

Supplementary Material

## Supplementary Figures





**Figure S1.** Trend of *ORs* and AIC according to the variation of model complexity for man.

**

**

**Figure S2.** Trend of *ORs* and AIC according to the variation of model complexity for woman.


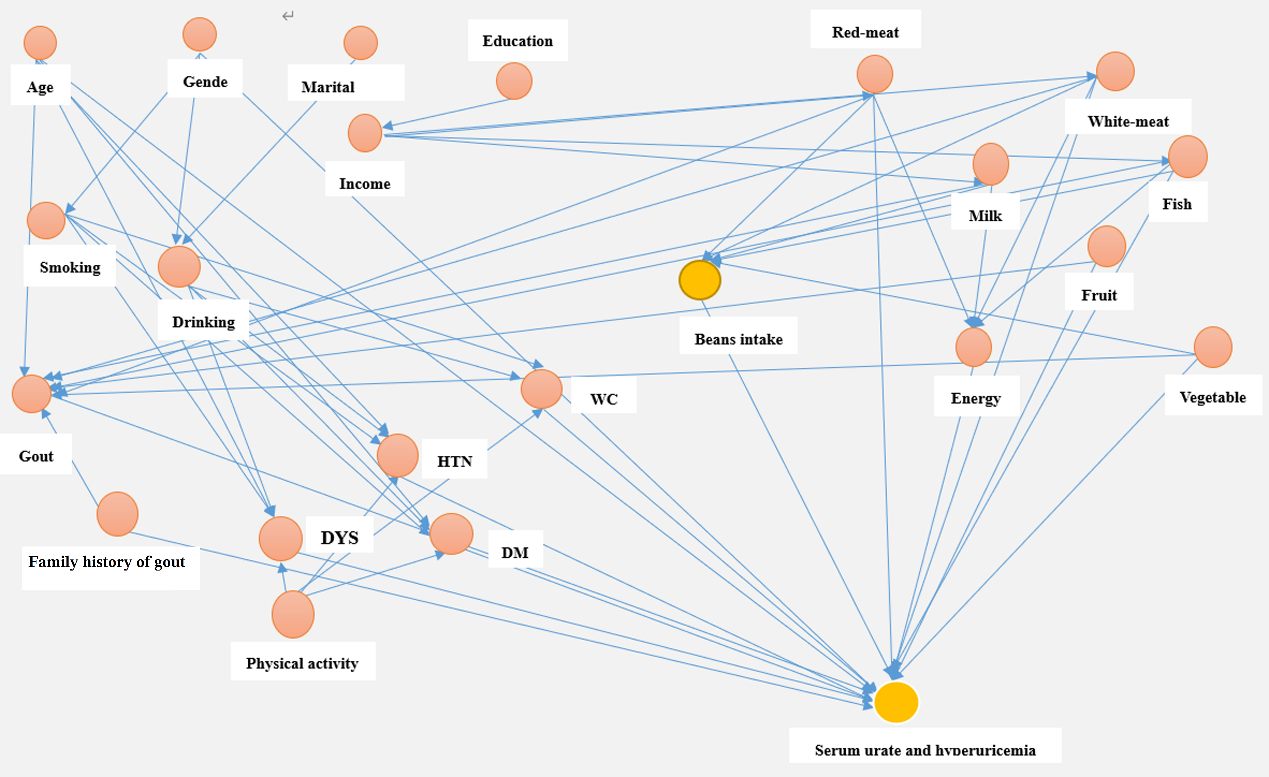


**Figure S3.** Directed acyclic graph for the association between beans intake and serum urate and hyperuricemia, showing all potential confounders.

| **Table S1 Correlation of serum urate with variables** | | | | | |
| --- | --- | --- | --- | --- | --- |
| **Variables** | **Men** | |  | **Women** | |
|  | **correlation coefficients** | ***P*** |  | **Kendall’s tau_b** | ***P*** |
| Age, (years) | -0.238 | < 0.001 |  | 0.068 | < 0.001 |
| Smoking status | -0.013 | 0.034 |  | 0.005 | 0.363 |
| Drinking status | 0.095 | < 0.001 |  | -0.006 | 0.236 |
| Physical activity | -0.041 | < 0.001 |  | -0.032 | < 0.001 |
| Education | 0.095 | < 0.001 |  | -0.023 | < 0.001 |
| Marital status | -0.039 | < 0.001 |  | 0.022 | < 0.001 |
| Average monthly individual income | 0.062 | < 0.001 |  | -0.008 | 0.107 |
| DYS | 0.167 | < 0.001 |  | 0.178 | < 0.001 |
| Diabetes mellitus | -0.095 | < 0.001 |  | -0.006 | 0.240 |
| Hypertension | 0.058 | < 0.001 |  | 0.092 | < 0.001 |
| Gout | 0.037 | < 0.001 |  | 0.001 | 0.834 |
| Family history of gout | 0.002 | 0.772 |  | -0.009 | 0.090 |
| WC | 0.273 | < 0.001 |  | 0.239 | < 0.001 |
| Red-meat (g/day) | 0.090 | < 0.001 |  | -0.004 | 0.557 |
| White-meat (g/day) | 0.077 | < 0.001 |  | 0.030 | < 0.001 |
| Fish (g/day) | 0.128 | < 0.001 |  | 0.053 | < 0.001 |
| Vegetable intake (g/d) | 0.044 | < 0.001 |  | 0.073 | < 0.001 |
| Fruit intake (g/d) | 0.041 | < 0.001 |  | 0.064 | < 0.001 |
| Energy intake ( kJ) | -0.009 | 0.254 |  | 0.002 | 0.737 |

DYS: dyslipidemia, WC: waist circumference. Correlation coefficients were calculated by Kendall’s tau_b analysis for categorical variable and Pearson analysis for continuous variable.

| **Table S2 Bivariate logistic regression of risk factors associated with hyperuricemia.** | | | | | |
| --- | --- | --- | --- | --- | --- |
| **Variables** | **Men** | |  | **Women** | |
|  | ***OR* (95% *CI*)** | ***P*** |  | ***OR* (95% *CI*)** | ***P*** |
| Age, (years) | 0.961 (0.958, 0.965) | < 0.001 |  | 1.106 (1.012, 1.020) | < 0.001 |
| Smoking status |  |  |  |  |  |
| Ex-smoker vs Nonsmoker | 0.964 (0.842, 1.103) | 0.596 |  | 2.251 (0.765, 6.623) | 0.141 |
| Smoker vs Nonsmoker | 0.984 (0.883, 1.096) | 0.763 |  | 0.906 (0.363, 2.260) | 0.833 |
| Drinking status |  |  |  |  |  |
| Ex-drinker vs Nondrinker | 1.034 (0.871, 1.227) | 0.704 |  | 0.759 (0.275, 2.096) | 0.595 |
| Drinker vs Nondrinker | 1.728 (1.562, 1.912) | < 0.001 |  | 0.828 (0.607, 1.129) | 0.232 |
| Physical activity |  |  |  |  |  |
| Moderate vs Low | 0.905 (0.806, 1.017) | 0.094 |  | 0.825 (0.743, 0.917) | < 0.001 |
| High vs Low | 0.728 (0.651, 0.825) | < 0.001 |  | 0.749 (0.662, 0.848) | < 0.001 |
| Education (≥ Junior school vs ≤ Primary school) | 1.608 (1.443, 1.791) | < 0.001 |  | 0.805 (0.734, 0.883) | < 0.001 |
| Marital status (Widowed/single/divorced/separated vs Married/cohabiting) | 0.751 (0.632, 0.892) | 0.001 |  | 1.303 (1.135, 1.495) | < 0.001 |
| Average monthly individual income |  |  |  |  |  |
| 500-1000 RMB vs < 500 RMB | 1.162 (1.031, 1.309) | 0.014 |  | 0.737 (0.659, 0.824) | < 0.001 |
| ≥ 1000 RMB vs < 500 RMB | 1.439 (1.283, 1.614) | < 0.001 |  | 0.910 (0.816, 1.015) | 0.089 |
| DYS (Yes vs No) | 2.570 (2.332, 2.831) | < 0.001 |  | 2.614 (2.382, 2.868) | < 0.001 |
| Diabetes mellitus (Yes vs No) | 0.594 (0.488, 0.723) | < 0.001 |  | 1.257 (1.088, 1.451) | 0.002 |
| Hypertension (Yes vs No) | 1.524 (1.384, 1.679) | < 0.001 |  | 1.949 (1.777, 2.137) | < 0.001 |
| Gout (Yes vs No) | 5.424 (3.237, 9.089) | < 0.001 |  | 0.762 (0.333, 1.748) | 0.522 |
| Family history of gout (Yes vs No) | 1.456 (0.418, 5.070) | 0.555 |  | 0.531 (0.166, 1.695) | 0.285 |
| WC, (cm) | 1.056 (1.051, 1.061) | < 0.001 |  | 1.054 (1.049, 1.058) | < 0.001 |
| Red-meat (g/day) | 1.005 (1.004, 1.006) | < 0.001 |  | 0.999 (0.977, 1.000) | 0.088 |
| White-meat (g/day) | 1.009 (1.007, 1.012) | < 0.001 |  | 1.003 (1.000, 1.006) | 0.043 |
| Fish (g/day) | 1.044 (1.036, 1.052) | < 0.001 |  | 1.013 (1.004, 1.022) | 0.004 |
| Vegetable intake (g/d) | 1.000 (1.000, 1.000) | 0.254 |  | 1.001 (1.000, 1.001) | < 0.001 |
| Fruit intake (g/d) | 1.000 (1.000, 1.001) | 0.038 |  | 1.001 (1.000, 1.001) | 0.001 |
| Energy intake ( kJ/d) | 1.000 (1.000, 1.000) | 0.578 |  | 1.000 (1.000, 1.000) | 0.648 |

CI: confidence interval, DYS: dyslipidemia, OR: odds ratio, WC: waist circumference.
